# Robust acute myeloid leukemia engraftment in humanized scaffolds using injectable biomaterials and intravenous xenotransplantation

**DOI:** 10.1002/1878-0261.13790

**Published:** 2025-01-22

**Authors:** Daniel Busa, Zdenka Herudkova, Jan Hyl, Jakub Vlazny, Filip Sokol, Kvetoslava Matulova, Adam Folta, Jakub Hynst, Lucy Vojtova, Leos Kren, Martin Repko, Zdenek Racil, Jiri Mayer, Martin Culen

**Affiliations:** ^1^ Department of Internal Medicine, Hematology and Oncology, Faculty of Medicine Masaryk University Brno Czech Republic; ^2^ Department of Pathology University Hospital Brno Czech Republic; ^3^ Department of Pathology, Faculty of Medicine Masaryk University Brno Czech Republic; ^4^ Department of Internal Medicine, Hematology and Oncology University Hospital Brno Czech Republic; ^5^ Central European Institute of Technology Masaryk University Brno Czech Republic; ^6^ Central European Institute of Technology Brno Institute of Technology Czech Republic; ^7^ Orthopedic Clinic University Hospital Brno Czech Republic; ^8^ Department of Orthopedic Surgery, Faculty of Medicine Masaryk University Brno Czech Republic; ^9^ Department of Physiology, Faculty of Medicine Masaryk University Brno Czech Republic

**Keywords:** AML, collagen, mouse model, ossicles, patient‐derived xenografts, T‐cell

## Abstract

Patient‐derived xenografts (PDXs) can be improved by implantation of a humanized niche. Nevertheless, the overall complexity of the current protocols, as well as the use of specific biomaterials and procedures, limits the wider adoption of this approach. Here, we identify the essential minimum steps required to create the humanized scaffolds and achieve successful acute myeloid leukemia (AML) engraftment. We compared seven biomaterials, which included both published and custom‐designed materials. The highest level of bone marrow niche was achieved with extracellular matrix gels and custom collagen fiber, both of which allowed for a simple non‐surgical implantation. The biomaterial selection did not influence the following AML infiltration. Regarding xenotransplantation, standard intravenous administration produced the most robust engraftment, even for two out of four otherwise non‐engrafting AML samples. In contrast, direct intra‐scaffold xenotransplantation did not offer any advantage. In summary, we demonstrate that the combination of an injectable biomaterial for scaffold creation plus an intravenous route for AML xenotransplantation provide the most convenient and robust approach to produce AML PDX using a humanized niche.

## Graphical abstract

Patient‐derived xenografts (PDXs) can be improved by implantation of a humanized niche. We tested different biomaterials and approaches, and demonstrate that the combination of an injectable biomaterial for scaffold creation plus an intravenous route for acute myeloid leukemia (AML) xenotransplantation provide the most convenient and robust approach to produce AML PDXs using a humanized niche, even for otherwise non‐engrafting samples.

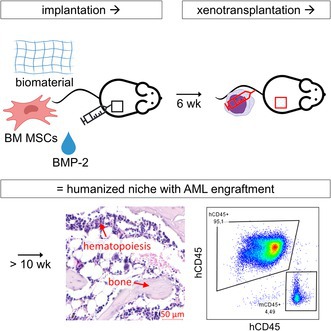

**Abbreviations**
AML: acute myeloid leukemia
BM: bone marrow
BMP‐2: bone morphogenetic protein 2
ECM: extracellular matrix
EDTA: ethylenediaminetetraacetic acid
GVHD: graft versus host disease
h: human
HA: hydroxyapatite
hPL: human platelet lysate
i.o.: intra‐osseous
i.sc.: intra‐scaffold
i.v.: intravenous
MSC: mesenchymal stromal cells
NSG: NOD.Cg‐*Prkdc* ^*scid*^ *Il2rg* ^*tm1Wjl*^/SzJ
NSGS: NOD.Cg‐*Prkdc* ^*scid*^ *Il2rg* ^*tm1Wjl*^/Tg(CMV‐IL3,CSF2,KITLG)1Eav/MloySzJ
PB: peripheral blood
PDX: patient‐derived xenograft
VAF: variant allele frequency
β‐TCP: β‐tricalcium phosphate

## 1 Introduction

A humanized niche that can form ectopic bone with bone marrow cavities in mice can be created by implanting a suitable biomaterial and human bone marrow (BM) mesenchymal stromal cells [1]. This approach can improve normal and malignant hematopoiesis engraftment in mice, even for less‐aggressive acute myeloid leukemia (AML) or other poorly engrafting hematologic malignancies, such as multiple myeloma and myeloproliferative neoplasms [2, 3, 4, 5, 6, 7]. A crucial factor in creating the humanized niche is the use of mesenchymal stromal cells (MSCs), specifically from BM, whereas MSCs from other tissues—adipose tissue, umbilical cord, and skin—fail to provide ossification *in vivo* [1]. A wide variety of applicable biomaterials have been described so far, including artificial and natural materials developed either by academia or available commercially—polyurethane scaffolds, ceramic calcium phosphate, extracellular matrix (ECM) gels, hydrogels, and gelatine or collagen meshes [2, 8, 9, 10, 11]. Scaffold creation may range from single‐ to multi‐step protocols. Simple procedures implant a biomaterial together with MSCs, while more complex ones pre‐seed MSCs on the biomaterial *in vitro*, several days before implantation [1, 7, 12], either with or without additional osteo‐ or chondro‐genic differentiation [5, 12, 13]. Malignant or normal blood cell xenotransplantation usually follows 6–8 weeks post‐implantation, when the scaffolds have already developed the BM niche. The cells can be delivered through a standard intravenous (i.v.) route, but direct intra‐scaffold (i.sc.) xenotransplantation was shown to provide faster engraftment [5]. Combining implantation and xenotransplantation into one step was also shown to produce improved engraftment, as well as the obvious time reduction [6, 7, 11]. In general, wider application of this protocol is now limited by methodological difficulty and the large differences between the published protocols.

This study aimed to compare various materials and methodologies to establish a robust and uncomplicated protocol for scaffold‐based AML patient‐derived xenografts (PDX). Initially, we evaluated the formation of a humanized niche using seven different biomaterials. Subsequently, we compared AML engraftment efficacy for four selected biomaterials. Finally, we compared different xenotransplantation routes and challenged the model with non‐engrafting AML samples using two most promising biomaterials.

## 2 Materials and methods

### 2.1 Donors and patients

MSCs were obtained from healthy donors undergoing total knee or hip endoprosthesis at the Orthopedic Clinic, University Hospital Brno, between. The BM was collected as a residual material during surgery.

Primary AML samples were collected at the time of diagnosis from patients treated at the Department of Internal Medicine – Hematology and Oncology, University Hospital Brno. Donor and patient characteristics are provided in Table S1.

The samples were collected between January 2016 and December 2021. All donors and patients provided written informed consent and the study was approved by the local ethical committee at the University Hospital Brno (donors – no. 01‐200116/EK, patients – no. 01‐270618/EK), according to the Helsinki Declaration.

### 2.2 BM MSC isolation and cultivation

BM (10–20 mL) was collected on Heparin (10 IU Hep per 1 mL BM) and diluted with phosphate‐buffered saline. The material was filtered through a sterile gauze and then through a 100 μm cell strainer. Leukocytes were obtained by red blood cell lysis with ammonium chloride solution and then seeded into culture vessels resuspended in α‐modified minimum essential medium (cat. no. M4526, Sigma‐Aldrich, St. Louis, MO, USA) with 5% human platelet lysate (hPL, obtained from platelets purchased from the Department of Transfusion and Tissue Medicine, University Hospital Brno, Brno, Czech Republic, according to the protocol by Reinisch et al. [9]). Non‐adherent cells were discarded on day 3, adherent cells were further cultured. The medium was changed twice a week until the cells reached approximately 90% confluence. This yielded freshly isolated passage zero cells that were passaged and expanded. The obtained passage 1 cells were cryopreserved or further cultured. Passage 2 or 3 cells were used for scaffold generation and the last passage/expansion was performed before implantation, so the cells were implanted fresh from the culture. The osteogenic capacity of MSCs from each donor and each passage was confirmed *in vivo* (data not shown), as *in vitro* differentiation capacity is not always indicative of *in vivo* bone forming capacity [1].

### 2.3 Biomaterials and preparation

The following biomaterial categories and their subtypes were prepared as follows:

1. β‐tricalcium phosphate (β‐TCP) ceramic granules with 0.5–1 mm size, Kasios® TCP Dental HP (cat. no. K4070550HP, Kasios, L'union, France). Approximately 100 μL of granules were mixed with MSCs resuspended in 20 μL of hPL and 80 μL of “Cultrex ECM gel” (Cultrex™ 3‐D culture matrix, cat. no. 3445, Bio‐Techne, Minneapolis, MN, USA). This yielded a slurry which could be surgically implanted using a spatula.
2. Three subtypes of ECM gels obtained from Engelbreth‐Holm‐Swarm mouse tumor were used: “Merck ECM gel” (cat. no ECM625, *In Vitro* Angiogenesis Assay Kit, Merck, Darmstadt, Germany), “Sigma ECM gel” (cat. no. E1270, ECM gel from Engelbreth‐Holm‐Swarm murine sarcoma, Sigma‐Aldrich), and the Cultrex ECM gel. For all ECM gels, 240 μL was mixed with MSCs resuspended in 60 μL of hPL and implanted by injection.
3. Collagen‐hydroxyapatite (HA) matrix prepared from a 0.5 wt % collagen solution and supplemented with 50 wt % HA, in the form of approximately 4 × 8 mm cylinders, prepared at CEITEC BUT, Brno, Czech Republic, as described previously [14]. The unmanipulated dry matrices were surgically inserted under the skin and only then immediately injected with MSCs resuspended in 40 μL of α‐MEM and 20 μL of hPL.
4. Collagen fibers were prepared as an injectable version of the collagen matrix that was ground with mortar and pestle after freezing with liquid nitrogen, at CEITEC BUT. The subtypes differed in calcium phosphate supplementation (none/HA/TCP). Ten milligrams of material were mixed with MSCs resuspended in 160 μL of α‐MEM and 40 μL of hPL. Within a few minutes, the material slightly gelled. Fibers with HA or TCP were implanted by injection. Fibers without HA/TCP had to be implanted surgically as their swelling in medium prohibited injection.

All collagen‐based materials were sterilized by ethylene oxide gas. Recombinant human/murine/rat bone morphogenetic protein 2 (BMP‐2) (cat. no. 120‐02, Peprotech, Rocky Hill, NJ, USA) was added to the scaffold mixture at preparation, if indicated. For most of the materials, multiple batches were used throughout the project: 1 for ceramic granules, 4 for Sigma ECM gel, 2 for Merck ECM gel, 1 for Cultrex ECM gel, 2 for collagen matrix, 1 for collagen‐noHA fiber, 3 for collagen‐HA fiber.

### 2.4 Animals, implantation, and xenotransplantation

Immunodeficient mice NOD.Cg‐*Prkdc* ^*scid*^ *Il2rg* ^*tm1Wjl*^/SzJ (NSG) or NOD.Cg‐*Prkdc* ^*scid*^ *Il2rg* ^*tm1Wjl*^/Tg(CMV‐IL3,CSF2,KITLG)1Eav/MloySzJ (NSGS) (The Jackson Laboratory, Bar Harbor, ME, USA) were kept in isolated ventilated cages under specific pathogen‐free conditions in an air‐conditioned room, at 18–23 °C, humidity 40–60%, with a 12‐h day/night cycle, and food and water provided *ad libitum*. Five to eleven (median 7) week old animals were used for scaffold implantation. The animal age was not found to affect scaffold creation (Fig. S1). For scaffold formation experiments without AML, animals of both sexes were chosen randomly (Table S2). For AML xenotransplantation experiments, single or equivalently mixed sexes were used to limit inter‐mouse heterogeneity (Table S2), since animal sex can influence engraftment [15].

All mouse experiments were performed in accordance with institutional guidelines and approved by the institutional Expert committee for ensuring the welfare of experimental animals of the Faculty of Medicine, Masaryk University and by the expert committee of the Ministry of Education, Youth and Sport of the Czech Republic – project licenses MSMT‐11783/2014‐5, MSMT‐21123/2018‐8, MSMT‐14930/2021‐4.

Implantation was usually performed 30 min after biomaterial preparation. The scaffolds were subcutaneously implanted into animals anesthetized by intraperitoneal etomidate injection (22 mg·kg^−1^). One to three separate scaffolds were implanted per mouse, on the animal's flanks or back. Surgical implantation involved inserting the scaffold through an approximately 5 mm long incision into a subcutaneous pocket. The wound was closed with one or two reflex clips. Injection implantation was performed through an 18‐gauge needle. After 4–8 weeks, the animals were either sacrificed for ossicle analysis or injected with AML cells and further followed.

AML was xenotransplanted as 0.4–1.0 × 10^6^ leukocytes, by i.v. or intra‐scaffold i.sc. injection or mixed with MSCs (mix). To prevent T‐cell expansion, the cells were treated with anti‐CD3 antibody OKT3 (cat. no. 40‐0037, Tonbo Biosciences, San Diego, CA, USA), 1 μg/1 × 10^6^ cells, either 15–30 min before injection for i.v. and i.sc. route (no wash), or 15 min for mix administration (wash) [16]. No BM conditioning, e.g., irradiation was used.

To monitor the engraftment in PB, regular tail vein bleedings were started 10 weeks from xenotransplantation. Subsequent intervals, from 1 to 4 weeks, were chosen based on the specific sample and engraftment progression.

Mice xenotransplanted with the well‐engrafting samples (Sections 3.2 and 3.3) were sacrificed when median engraftment in PB reached ≥ 1% in all mice for a specific AML sample. Mice with the non‐engrafting samples (Section 3.5) were sacrificed at 24 weeks, T‐lymphocyte detection or signs of graft‐versus‐host disease.

### 2.5 Sample processing and flow cytometry analysis

Murine PB (50–70 μL) was aspirated into an 0.5 mL insulin syringe (cat. no. 9151125S, B. Braun Medical, Hessen, Germany) containing 50 μL of 0.02 mm ethylenediaminetetraacetic acid (EDTA) solution in DPBS without calcium and magnesium (cat. no. D8537, Merck, Burlington, MA, USA) and then transferred to a blood EDTA collection tube (cat. no. 1501126, Aquisel, Abrera, Spain). BM from murine femurs was obtained by quick centrifugation [17], resuspended in a 1 mL of DPBS with 0.02 mm EDTA and filtered through a 30 μL cell strainer (cat. no. 04‐0042‐2316, Sysmex, Tokyo, Japan). Scaffolds were gently crushed using a mortar and pestle, washed with 1 mL of DPBS +0.02 EDTA solution and filtered.

Human BM from sternal puncture was analyzed using lyse‐stain‐wash protocol, murine material using stain‐lyse‐wash protocol.

Murine PB from regular bleedings was analyzed with a 3‐color tube: 7‐Aminoactinomycin D (cat. no. A1310, Thermo Fisher Scientific, Waltham, MA, USA), murine CD45 APC (clone 30‐F11, cat. no. 1115560), human CD45 BV421 (clone HI30, cat. no. 2120160), and in case of lymphocyte detection also with human CD3 BV510 (clone UCHT1, cat. no. 2102240) (all antibodies from Sony Biotechnology, San Jose, CA, USA).

Human BM and murine PB, BM, and scaffolds from final analysis were analyzed with an 8‐color tube: 7‐Aminoactinomycin D, murine CD45 APC, and human CD45 BV421, CD34 FITC (clone 581, cat. no. 2317520), CD38 PE/Cy7 (clone HB‐7, cat. no. 2383040), CD33 PE (clone P67.6, cat. no. 2433040), CD14 APC‐Cy7 (clone M5E2, cat. no. 2109100), and CD3 BV510 (all Sony Biotechnology).

The analyses were performed on a BD FACS Verse instrument (BD Biosciences, San Jose, CA, USA). At final analysis, AML engraftment was assessed in samples with ≥ 50 live single cells and was considered positive in cases with more than 1% hCD45^+^Lym^−^ cells or hCD45^+^ cells expressing blast/myeloid markers CD33, CD34, or CD14. Fraction of CD34^+^ or CD34^+^CD38^−^ cells was assessed only in samples with ≥ 50 hCD45^+^ cells.

### 2.6 Histological and image analysis

Extracted scaffolds and murine femurs were fixed in 4% paraformaldehyde. Decalcification was performed with EDTA solution. Hematoxylin and eosin staining, and immunohistochemistry staining of human Vimentin (clone V9, Leica Biosystems, Nussloch, Germany) and human CD34 (clone QBEnd 10, Dako, Jena, Denmark) antibodies was performed using standard protocols.

Histological slides were scanned using an Axio Scan.Z1 instrument and ZEN 2.6 (blue edition) software (Carl Zeiss Microscopy, Jena, Germany). Images were exported to Tagged Image File Format (tiff). Image analysis was performed with imagej's sfiji (https://fiji.sc). The following regions of interest were selected using a freehand or wand tool and then quantified: bone segments, areas with hematopoietic or leukemic colonization, empty areas from decalcified and washed‐out β‐TCP granules.

### 2.7 Next‐generation sequencing

The libraries were prepared with a 37‐gene targeted panel VariantPlex® Core Myeloid Kit (Integrated DNA Technologies, Coralville, IA, USA), except for 2 original AML samples (Table S1). *FLT3‐ITD* mutations were additionally analyzed with in‐house fragment analysis. The subsequent in‐house bioinformatic analysis was developed with special attention to handle contaminating mouse reads. *In silico* synthetic mouse (GRCm38.p6‐93) and human (GRCh37‐p13) genomic references were used in the read mapping step. Sequencing reads mapped only to the mouse reference were ignored in the variant calling step. All software used in the bioinformatic analysis pipeline is listed in Table S3. The pipeline is freely available at https://github.com/Hynst/mix_mouse_human_pipeline.

Amplicon sequencing of *WT1* gene mutations was performed as a two‐step PCR with Platinum SuperFi II PCR Master Mix and Platinum SuperFi II Polymerase (both Thermo Fisher Scientific). The first PCR was performed with 10 μL of isolated DNA (100–700 ng per reaction) and primers WT1‐F (TCGTCGGCAGCGTCAGATGTGTATAAGAGACAGCTCTCTGCCTGCAGGATGTG) and WT1‐R (GTCTCGTGGGCTCGGAGATGTGTATAAGAGACAGGGCGTTTCTCACTGGTCTCA) defining the region where *WT1* variants are located. The PCR protocol was 98 °C for 1 min, 10 cycles of 98 °C 10 s, 60 °C 10 s and 72 °C 20 s, followed by 72 °C for 5 min. The PCR product was purified by SPRI Select beads (Beckman Coulter, Brea, CA, USA) and used for second PCR with Illumina Nextera indexes. The cycling conditions were the same as the first PCR, with only 20 cycles. The PCR products were purified using SPRI Select beads, quantified using Qubit dsDNA High Sensitivity Kit (cat. no. Q33230, Thermo Fisher Scientific) and pooled for sequencing on Nextera platform (Illumina, San Diego, CA, USA).

All sequencing was performed on the NextSeq platform (Illumina).

### 2.8 Statistics and data availability

Statistical analyses were performed using GraphPad Prism 8.0 (GraphPad Software, Boston, MA, USA), except for power analysis which was estimated by applying 1000 bootstrap replicates for each test, using R programming. A *P*‐value < 0.05 was considered statistically significant. Details and results of all statistical analyses are listed in Table S2, along with the data graphed in individual figures.

## 3 Results

### 3.1 Biomaterials differ in BM niche formation

**Fig. 1:**
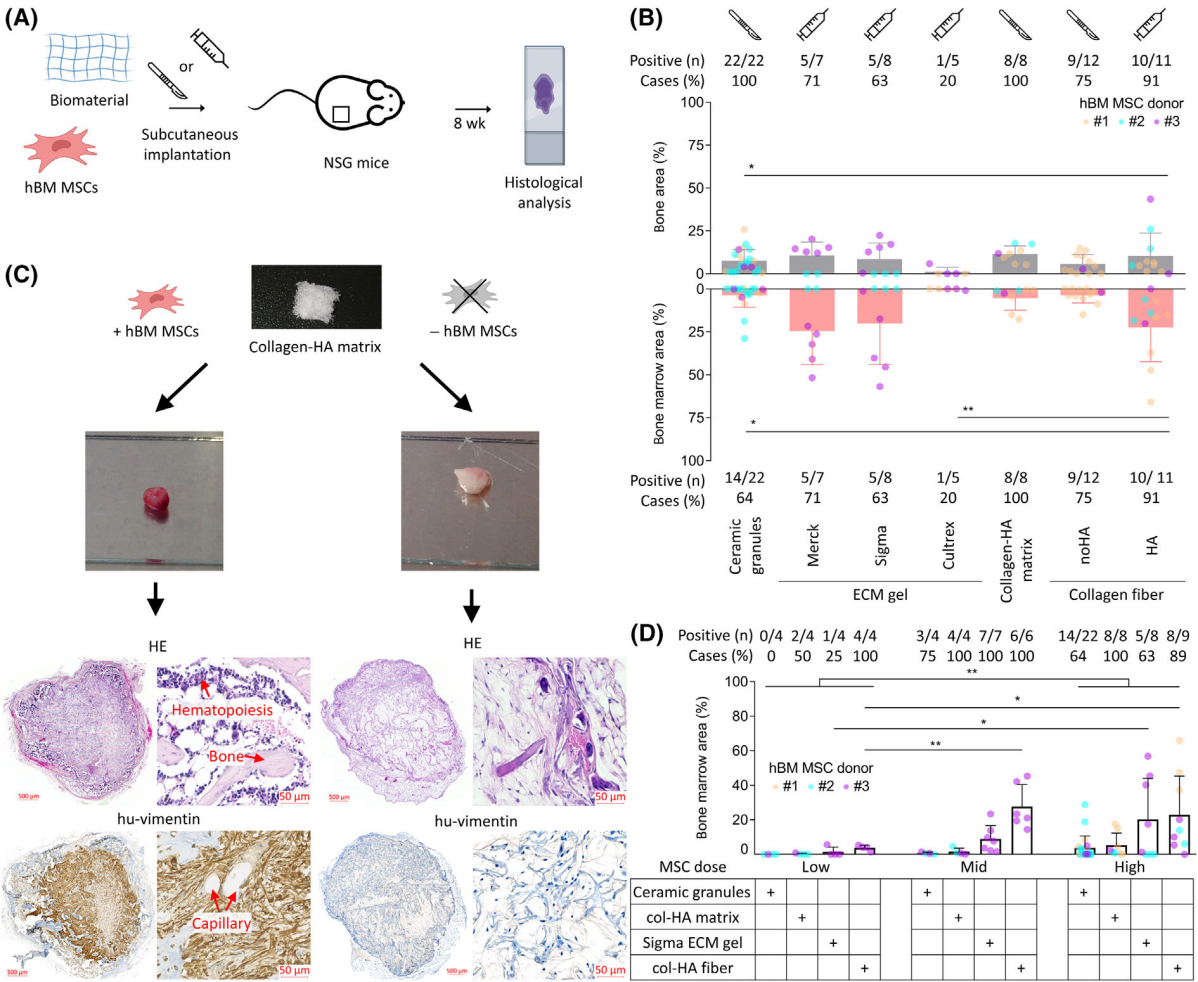
Generation of scaffolds with different biomaterials. (A) Schematic workflow. (B) Evaluation of bone and bone marrow formation in scaffolds created from different biomaterials. Area of mature bone trabeculae and hematopoiesis quantified using image analysis of histological slides. (unpaired, non‐parametric one‐way ANOVA). Scalpel or syringe icon denotes implantation route—surgical or injection, respectively. Scaffolds generated with 2 × 10^6^ s passage BM MSCs. (C) Example of bone and bone marrow formation. Left—scaffold implanted with MSCs, right—control scaffold implanted without MSCs. From top: collagen‐HA matrix before implantation and then after extraction from mice (approximately in the same scale); visualization of bone trabeculae and BM niche areas with hematoxylin and eosin (HE) staining; confirmation of the human origin of osteoblasts and the undifferentiated fibrous tissue inside the scaffolds with anti‐human vimentin staining. Scale bar = 500 μm for whole scaffolds, 50 μm for close‐up. (D) The effect of three different MSC doses on BM niche formation tested using 4 biomaterials, each representing one category (ordinary two way ANOVA). (B, D) Positive case defined as ≥ 0.1% bone or bone marrow area. Columns show mean values with standard deviation. Only statistically significant differences shown in the graphs. BM, bone marrow; col, collagen; ECM, extracellular matrix; h/hu, human; HA, hydroxyapatite; HE, hematoxylin and eosin; MSC, mesenchymal stromal cell; *n*, number; NSG, NOD.Cg‐*Prkdc* ^*scid*^ *Il2rg* ^*tm1Wjl*^/SzJ, *P < 0.05, **P < 0.01.

The first aim was to assess the bone and BM formation potential of 7 distinct biomaterials from 4 categories – (1) commercially available β‐TCP ceramic granules, (2) ECM gels from three different commercial manufacturers, (3) a novel “sponge‐like” collagen‐HA matrix of academic origin [14], and (4) a derived collagen fiber with or without hydroxyapatite (collagen‐HA and collagen‐noHA, respectively). The ceramic granules and collagen‐HA matrix were solid and required surgical implantation, while ECM gels and collagen fibers were semi‐liquid and could be implanted by injection (Fig. 1A,B). The exception was the collagen‐noHA fiber which could not be injected due to its intense swelling. The materials were implanted with human BM (hBM) MSCs, except for negative controls. Analysis by histological examination and image analysis followed after 8‐week incubation period (Fig. 1A) [9, 11, 18]. The experiments were performed over a longer time (6 years) to challenge the robustness of the procedure.

The bone trabeculae with osteoblasts were mostly formed on the scaffold peripheries, whereas the inner scaffold regions were often filled with fibrous tissue of human origin (Fig. 1C). The exception was the ceramic granules where bone formed around the granules throughout the whole scaffold (Fig. S2). The BM niche containing murine hematopoiesis was located mostly in cavities surrounded by bone segments. Vasculature and deep‐red BM niche cavities in successfully remodeled scaffolds were macroscopically visible upon extraction. Human‐specific vimentin staining confirmed human origin of the bone, osteoblast nuclei, and residual fibrous cells, while the hematopoiesis and endothelium stained negative (Fig. 1C). All control scaffolds without MSCs (tested with 3 × ceramic granules, 2 × collagen‐HA matrix, 3 × collagen‐HA fiber) remained white, with no bone formation or vimentin positivity, and were filled with reactive granulation tissue showing resorptive reaction (Fig. 1C, Fig. S2).

The bone was formed in 20–100% scaffolds, based on material subtype, with the best reproducibility observed with the ceramic granules and collagen‐HA matrix (Fig. 1B). The bone area extent was similar between materials, except for poorly performing Cultrex ECM gel. The BM niche with murine hematopoiesis characterized by the presence of tri‐linear hematopoiesis and adipocytes was observed only in scaffolds positive for bone formation (Fig. 1B). The best BM formation success rate and extent was observed with Merck and Sigma ECM gels, and collagen‐HA fiber. For the fibers, we also tested variants with or without collagen crosslinking and found that this made no difference to bone or BM niche formation (Fig. S3). As a result, both variants (cross/non‐crosslinked) were used interchangeably.

Additionally, we investigated MSC seeding dose and showed that the initial dose of 2 × 10^6^ P2 BM MSCs can be reduced by a half, while retaining sufficient BM niche formation (Fig. 1D).

Taken together, we showed that material selection affects reproducibility and BM niche formation and that the MSC dose can be titrated.

### 3.2 AML engraftment is not influenced by biomaterial

**Fig. 2:**
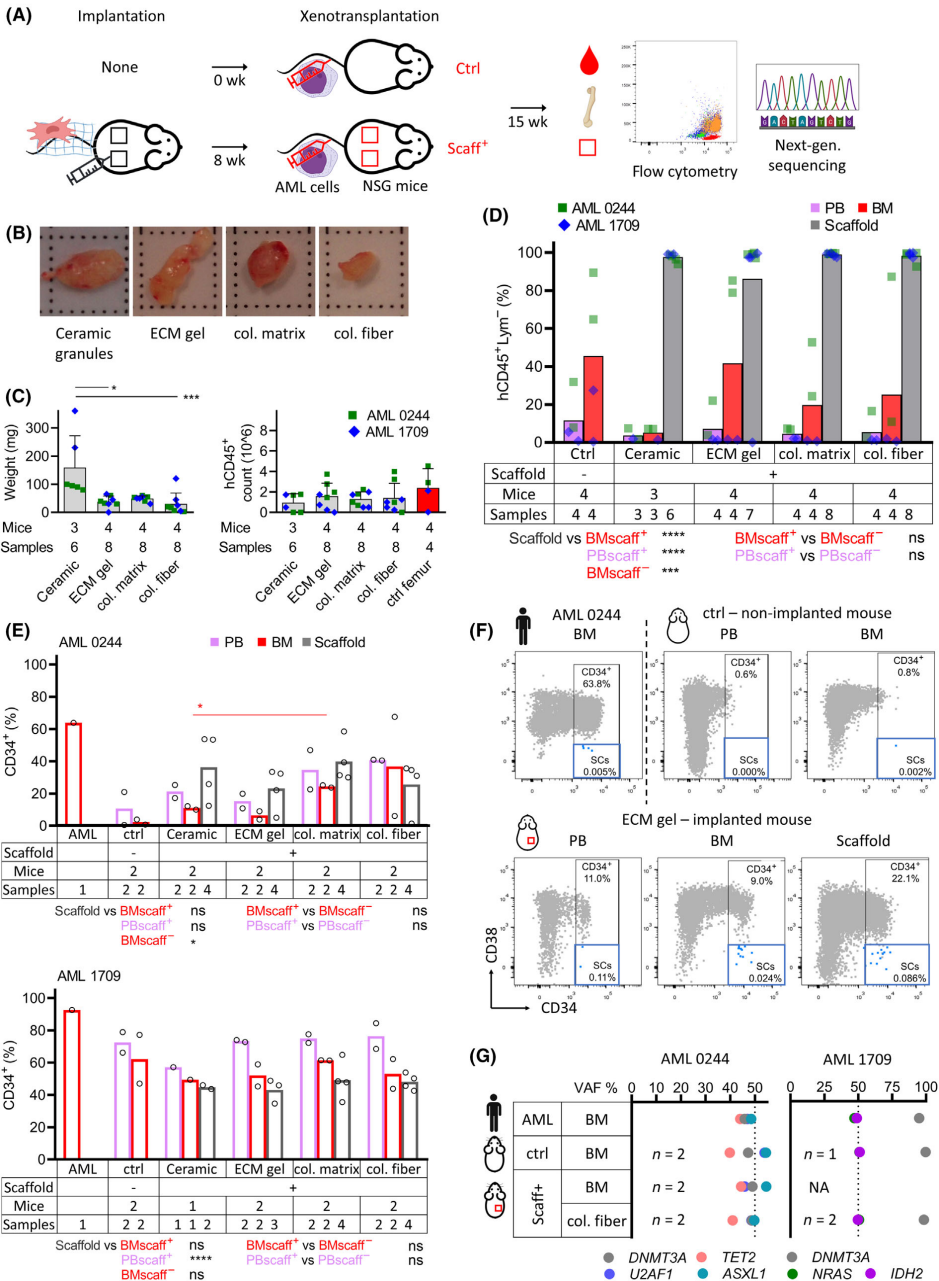
Comparison of 4 selected biomaterials in regard to AML engraftment – ceramic granules (ceramic), Sigma ECM gel (ECM gel), collagen‐HA matrix (col. matrix), collagen‐HA fiber (col. fiber). (A) Schematic workflow. (B) Photographs of extracted scaffolds infiltrated with AML (all > 90% hCD45^+^Lym^−^ cells, AML 0244). (C) Comparison of scaffold weights and yields of extracted human cells (unpaired, non‐parametric one‐way ANOVA). (D) AML engraftment levels in the xenotransplanted mice in different tissues (within‐between two‐way ANOVA, unpaired non‐parametric *t*‐test). (E) Percentage of CD34^+^ cells in the original AMLs and derived PDXs. Only samples with ≥ 50 hCD45^+^ cells analyzed (within‐between two way ANOVA, unpaired non‐parametric *t*‐test). (F) Example of CD34^+^ and CD34^+^CD38^−^ cell populations in: primary AML sample, scaffold^−^ control mouse, and scaffold^+^ mouse. (G) Mutation analysis of the original AML sample and pooled samples of selected mice (*n* denotes number of pooled mice). General info: Two scaffolds implanted per mouse. Scaffolds generated with 1 × 10^6^ s passage BM MSCs. AML 1709—one mouse from ceramic group died before analysis, one scaffold from ECM gel group resorbed. Columns in graphs show mean values, whiskers standard deviation. Only statistically significant differences shown in the graphs. AML, acute myeloid leukemia; BM, bone marrow; col., collagen; ctrl, control; ECM, extracellular matrix; MSC – mesenchymal stromal cell; *n*, number; NA, not available; ns, not significant; NSG, NOD.Cg‐*Prkdc* ^*scid*^ *Il2rg* ^*tm1Wjl*^/SzJ; PB, peripheral blood; PDX, patient‐derived xenograft; SC, stem cell; scaff, scaffold; VAF, variant allele frequency; wk, week, *P < 0.05, ***P < 0.001, ****P < 0.0001.

Next, we examined the effect of biomaterials on AML engraftment (Fig. 2A). We tested one material per category—ceramic granules, Sigma ECM gel (preferred due to slower gelling which facilitated injection), collagen‐HA matrix, and collagen‐HA fiber. Two well‐engrafting AML samples, known to engraft unmanipulated NSG mice (data not shown), were used.

At macroscopic observation, most of the extracted scaffolds appeared paler than without AML (Fig. 2B). Although the ceramic granules produced larger scaffolds by weight, the yield of extracted human cells was identical to other materials (Fig. 2C). Importantly, the AML engraftment did not differ between the materials, and all scaffolds reached full infiltration (Fig. 2D, Figs S4 and S5A,B). One Sigma ECM gel scaffold was resorbed and was thus assigned 0% engraftment. The engraftment in scaffolds was higher than in BM of the same mice and than in BM of scaffold^−^ controls. The femurs from scaffold^−^ mice yielded similar hCD45^+^ cells counts as scaffolds, but at a lower AML engraftment, meaning that the femurs housed higher absolute cell counts, human and murine together (Fig. 2C).

To evaluate the engraftment quality, we assessed the percentage of primitive CD34^+^ cells (Fig. 2E). No difference was observed between the biomaterials, but with AML 0244, more CD34^+^ cells were seen in scaffold^+^ than scaffold^−^ mice. Similarly, CD34^+^CD38^−^ cells were detectable in scaffold^+^ mice but almost absent or one log lower in scaffold^−^ mice, and interestingly also in the original AML sample (Fig. 2F and Fig. S6). To analyze possible clonal changes at the genetic level, we sequenced 37 genes (only for collagen‐HA fiber group; Table S4). This revealed no clonal skewing between the original AML samples, scaffold^−^ and scaffold^+^ mice (Fig. 2G).

Taken together, we saw no quantitative or qualitative differences in AML engraftment between the biomaterials. The AML preferentially populated the scaffolds over the murine BM and the scaffolds increased the percentage of primitive AML cells in murine PB and BM in one of two tested AML samples.

### 3.3 Systemic and intra‐niche xenotransplantation produce similar engraftment

**Fig. 3:**
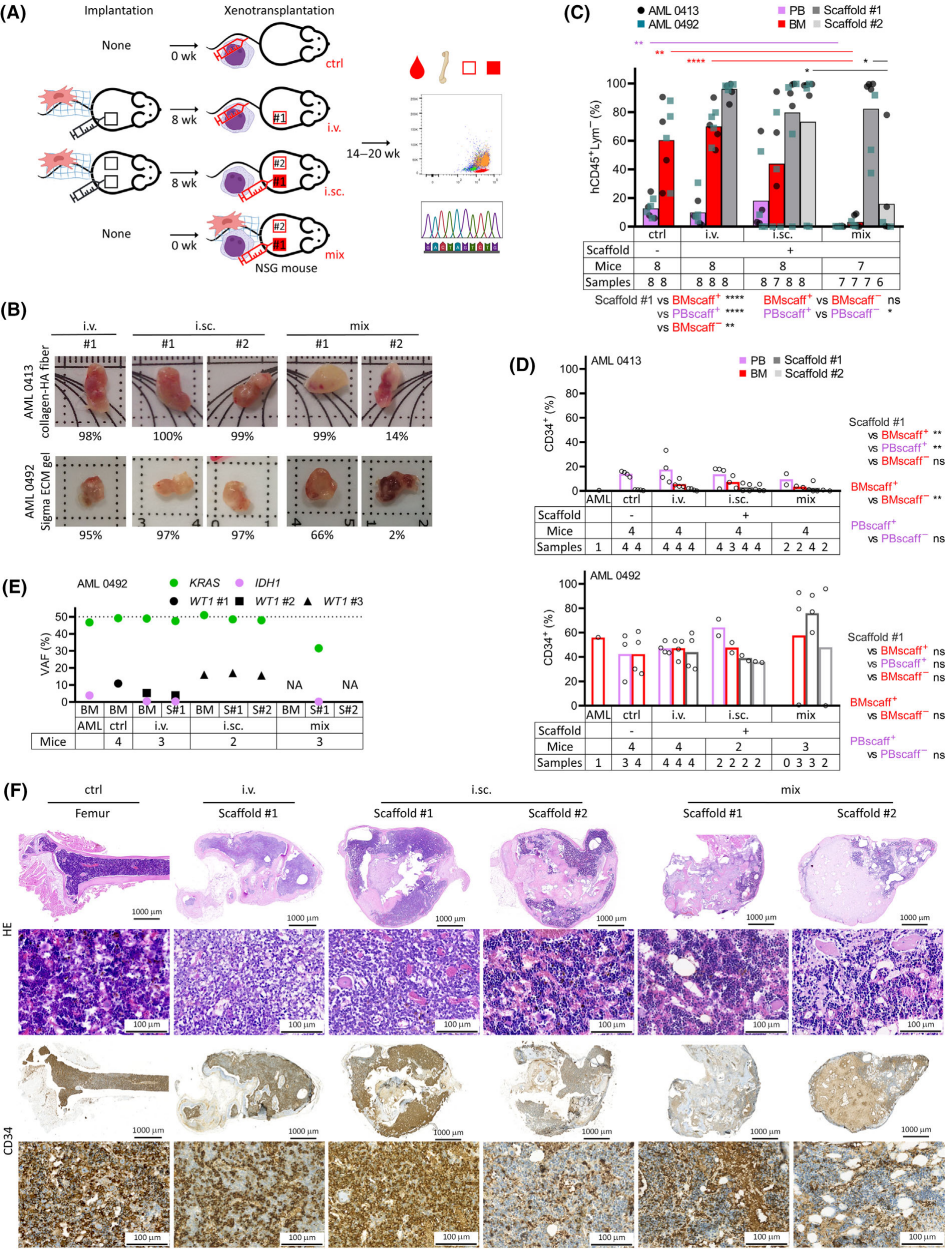
Comparison of xenotransplantation routes. (A) Experimental parameters and workflow. (B) Photographs of extracted scaffolds, with percentage of hCD45^+^Lym^−^ cells. (C) Engraftment in the xenotransplanted mice. AML 0413 (within‐between two way ANOVA, unpaired or paired non‐parametric *t*‐test). (D) Percentage of CD34^+^ cells in the original AML and derived PDXs. Only samples with ≥ 50 hCD45^+^ cells analyzed (within‐between two way ANOVA, unpaired or paired non‐parametric *t*‐test). (E) Mutation analysis of the original AML and pooled PDX samples in AML 0492. Number of mice pooled per each PDX group shown below the graph. Dotted line at 50% VAF indicates the maximum mutation load for heterozygous mutations, representing 100% mutated cells. (F) Histology of representative AML 0492 scaffolds. Approximate AML infiltration assessment: ctrl, i.v., and i.sc. #1 – high; i.sc. #2 – low; mix #1 and #2 – low/none. Scale bar = 1000 μm for whole scaffolds, 100 μm for close‐up. General info: Scaffolds generated with 1 × 10^6^ s passage BM MSCs, collagen‐TCP fiber used for AML 0413 and Sigma ECM gel for 0492. AML 0413—one BM from i.sc. group not analyzable by flow cytometry, AML 0492 – one scaffold #2 from mix group resorbed. Columns in graphs show mean values. Only statistically significant differences shown in the graphs. AML, acute myeloid leukemia; BM, bone marrow; ctrl, control; ECM, extracellular matrix; HA, hydroxyapatite; HE, hematoxylin and eosin; i.sc., intra‐scaffold; i.v., intravenous; MSC, mesenchymal stromal cell; *n*, number; N/A, not available; ns, not significant; NSG, NOD.Cg‐*Prkdc* ^*scid*^ *Il2rg* ^*tm1Wjl*^/SzJ; PB, peripheral blood; PDX, patient‐derived xenograft; PDX, patient‐derived xenograft; S, scaffold; scaff, scaffold; TCP, tricalcium phosphate; VAF, variant allele frequency; wk, week, *P < 0.05, **P < 0.01, ****P < 0.0001.

As a next step, we compared 3 different AML administration routes—the standard i.v. route, direct i.sc. injection, and adding the AML cells to the scaffold mixture at implantation (termed “mix”; Fig. 3A). The i.sc. and mix condition also contained a secondary AML‐unseeded scaffold to evaluate AML migration outside the AML‐seeded scaffold. Two well‐engrafting AML samples (different from the previous step) were chosen and tested with injectable materials, either with collagen‐TCP fiber (a newer modified version) or Sigma ECM gel.

There was no difference in the engraftment of the primary scaffolds between the injection routes (Fig. 3B,C, Fig. S7A,B). An engraftment failure was seen only in 1/8 scaffolds in the i.sc. group, probably due to technically failed intra‐scaffold AML injection. More prominent differences were seen in infiltration of murine tissues and the secondary AML‐unseeded scaffolds. The mix condition provided minimum AML migration as it failed to engraft 3/6 AML‐unseeded scaffolds (1 additional scaffold was resorbed) and provided little engraftment in the murine BM and peripheral blood (PB). The i.sc. condition failed to engraft the AML‐unseeded scaffolds in 2/8 and BM in 2/7 cases (1 additional BM could not be analyzed). The i.v. condition yielded BM engraftment in all mice, similar to the scaffold^−^ controls. Histological analysis confirmed that AML either completely occupied the BM cavities or mixed with the murine hematopoiesis in scaffolds with lower engraftment (Fig. 3F).

CD34^+^ cell frequency in the scaffolds did not differ between xenotransplantation routes (Fig. 3D). With AML 0413, the scaffold^+^ mice carried more CD34^+^ cells than scaffold^−^ controls in BM, with apparent peripheralization of the CD34^+^ from scaffolds to BM and even more into PB. Intense egress into the PB was also seen for CD34^+^CD38^−^ cells in both samples (Fig. S8).

Mutational analysis in AML 0492 revealed a subclonal evolution (Fig. 3E, Table S4). A dominant *KRAS* mutation was conserved in all murine samples but a low‐level *IDH1* mutation was mostly lost after xenotransplantation. Moreover, the xenografts produced three new *WT1* mutations, specific for each mouse group (pooled samples), showing a similar clonal evolution. An additional analysis of the original AML and individual mouse samples using a more sensitive amplicon analysis (up to 0.1% variant allele frequency, VAF) detected 4 *WT1* variants – 3/4 in the original AML (0.2–3.8% VAF) and 3/4 in the PDXs, one or two variants per group (Fig. S9). This showed that the PDX favored an isolated expansion of minor *WT1* ^pos^ subclones already existing in the primary sample.

In summary, the standard i.v. route provided robust engraftment in scaffolds and murine tissues. The more technically challenging i.sc. route did not provide any benefit. The mix route represented the simplest and fastest AML delivery but failed to produce engraftment in tissues outside the AML‐seeded scaffolds.

### 3.4 Scaffold bone marrow formation can be reduced to 6 weeks and enhanced with bone morphogenetic protein 2 (BMP‐2)

**Fig. 4:**
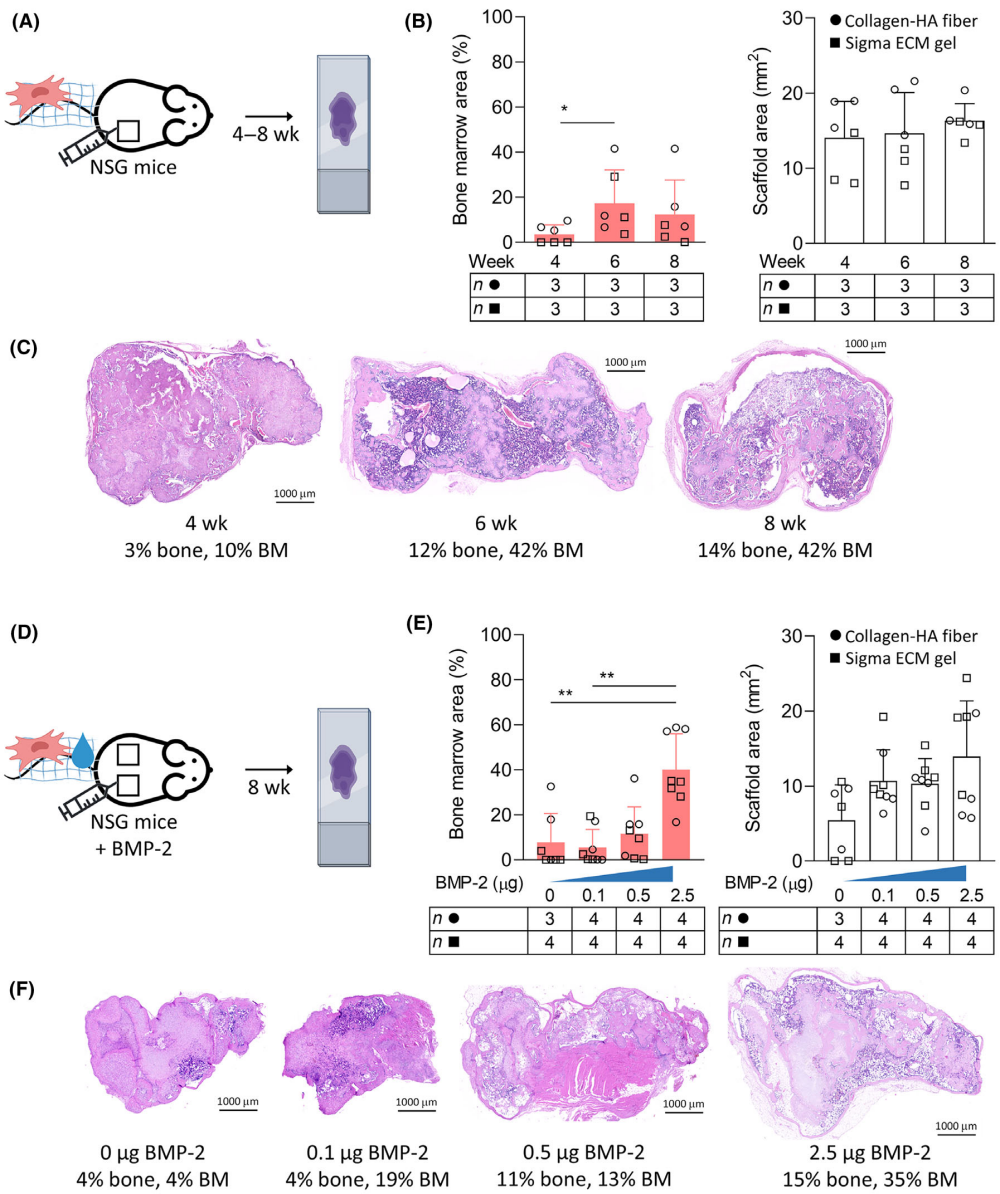
Optimization of scaffold development. (A–C) Dynamics of scaffold development – analysis at 4, 6, and 8 weeks after implantation. (A) Brief experimental workflow. One scaffold implanted per mouse. (B) Analysis of BM niche area and the overall scaffold size based on image analysis. (C) Examples of scaffolds from the individual time points. Collagen fiber scaffolds with the highest BM formation shown in HE staining. (D–F) The effect of increasing doses of BMP‐2 on scaffold formation. (D) Brief experimental workflow. Two scaffolds implanted per mouse. (E) Analysis of BM niche area and the overall scaffold size based on image analysis. One scaffold with 0 μg of BMP‐2 resorbed. (F) Examples of the scaffolds produced with different BMP‐2 doses. Sigma ECM gel scaffolds with the highest BM formation shown in HE staining. General info: Scaffolds generated with 1 × 10^6^ passage two BM MSCs. Columns in graphs show mean values with standard deviation. Only statistically significant differences shown in the graphs (unpaired, non‐parametric, one‐way ANOVA). Scale bar = 1000 μm. BM, bone marrow; BMP, bone morphogenetic protein; ECM, extracellular matrix; HA, hydroxyapatite; HE, hematoxylin and eosin; MSC, mesenchymal stromal cell; *n*, number; NSG, NOD.Cg‐*Prkdc* ^*scid*^ *Il2rg* ^*tm1Wjl*^/SzJ; wk, week, *P < 0.05, **P < 0.01.

To shorten the scaffold incubation period before xenotransplantation (valid for i.v. and i.sc. route), we investigated scaffold remodeling at 4, 6, and the standard 8 weeks post‐implantation (Fig. 4A). This revealed sufficient BM niche formation already at 6 weeks, with no further improvement at 8 weeks (Fig. 4B,C). At 4 weeks there was no/limited BM niche formation.

In parallel, we tried enhancing the scaffold formation via bone induction (Fig. 4D). Addition of 2.5 μg BMP‐2 at implantation not only increased BM niche formation but also more than doubled the overall scaffold size compared to 0 μg BMP‐2 controls, although the latter did not reach statistical significance (Fig. 4E,F).

The 6‐week incubation and BMP‐2 addition were combined in the following experiment.

### 3.5 Intravenous xenotransplantation route provides engraftment of non/low‐engrafting AML samples

**Fig. 5:**
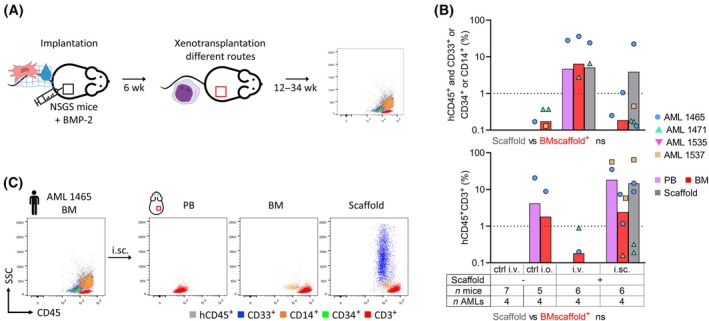
Scaffold testing with non/low‐engrafting AML samples. (A) Brief experimental workflow. (B) Engraftment in xenotransplanted mice. Upper graph shows AML engraftment, lower graph shows T‐cell expansion. Dotted line at 1% denotes positive engraftment threshold. Paired non‐parametric *t*‐test. (C) Immunophenotype example of the original AML and i.sc. PDX with T‐cell expansion. General info: Scaffolds generated with 2 × 10^6^ third passage BM MSCs; Sigma ECM gel used for AML 1699 and collagen‐TCP fiber used for the remaining samples. AML, acute myeloid leukemia; BM, bone marrow; BMP, bone morphogenetic protein; ctrl, control; ECM, extracellular matrix; GVHD, graft versus host disease; i.o., intra‐osseous; i.sc., intra‐scaffold; i.v., intravenous; MSC, mesenchymal stromal cell; *n*, number; NSGS, NOD.Cg‐*Prkdc* ^*scid*^ *Il2rg* ^*tm1Wjl*^/Tg(CMV‐IL3,CSF2,KITLG)1Eav/MloySzJ; PB, peripheral blood; TCP, tricalcium phosphate; wk, week.

As a final step, we tested if the scaffolds could support non‐engrafting AML samples (Fig. 5A). Since NSG non‐engrafting samples can engraft in the NSGS mouse strain, we switched to the NSGS mice and tested AML samples known to provide no or very limited engraftment even in this strain – 1 favorable, 2 intermediate, and 1 adverse prognostic risk (data not shown). We tested the i.v. versus the i.sc. xenotransplantation route and included intra‐osseous (i.o.) injection in scaffold^−^ mice as a negative control for the intra‐niche delivery.

Two out of four AML samples provided positive AML engraftment (≥ 1% of hCD45^+^ cells expressing CD33, CD34, or CD14) – one with i.v. and i.sc. route, the other only with i.v. route (Fig. 5B). The engraftment was mainly detected in the scaffolds, and at lower levels in the murine tissues. Despite OKT3 antibody pre‐treatment, we observed T‐cell expansion (≥ 1% hCD45^+^CD3^+^ cells) with two AMLs, but only with the intra‐niche routes – i.o. and i.sc. (Fig. 5B,C). Because of graft versus host disease (GVHD), the mice in this experiment were sacrificed earlier (11–24 weeks post xenotransplantation, except for i.v. scaffold^−^ controls) than the planned 30‐week observation interval. Because of this shorter monitoring time, we detected only a probably beginning AML engraftment (below the 1.0% threshold) in 4 i.o. and 4 i.sc. mice.

Taken together, the i.v. route supported the engraftment of 2/4 poorly engrafting samples. The i.sc. route provided engraftment in 1/4 and a possible starting engraftment in further 2/4 samples but produced a concurrent T‐cell expansion.

## 4 Discussion

Humanized microenvironment can be used as an upgrade in PDX models, however at the cost of increased experimental demands. It is now critical that the method can be easily reproduced and does not remain an exotic state‐of‐the‐art protocol. Here, we tested different approaches to scaffold creation and AML xenotransplantation to find a robust and easy‐to‐adopt protocol.

In biomaterial comparison, we assessed both commercial and academic‐origin materials. The commercial ones included ceramic β‐TCP granules as an alternative to previously described ceramic material [4, 5, 19], as well as ECM gel used by Reinisch et al., along with two other gels from different manufacturers [9]. The academic materials included collagen‐based spongous matrix and loose fibers, as a novel approach with potential for customization. A similar material supported osteogenesis in bone‐defect healing, suggesting possible utility in ossicle formation [20]. We opted not to include commercial gelatine matrices (Gelfoam®) due to the prior availability of the collagen materials and our preference for injectable materials [6]. Calcium phosphate materials and collagen I are well known to upregulate osteogenic genes and their products, such as runt‐related transcription factor 2—RUNX2, osteocalcin, osteopontin, and BMP 2/3 and to induce spontaneous ossification even in non‐osteogenic media [21, 22, 23]. This is in line with our findings, where nearly all scaffolds created from β‐TCP ceramic granules and collagen materials supplemented with HA or TCP showed bone formation. In the case of ECM gels, a multifactorial effect can be expected. These gels contain a complex mixture of structural proteins and growth factors and were shown to improve not only osteogenic but also chondro‐ and adipogenic differentiation [24, 25, 26]. Overall, our findings demonstrate variations in BM niche formation across the different biomaterials. However, this was not reflected in subsequent AML engraftment, as all materials exhibited full infiltration irrespective of their BM niche formation. Combined data for 4 well‐engrafting AML samples revealed that engraftment failure was less common (6/72, 8% scaffolds, Figs 2 and 3) than the BM niche formation failure without AML (12/49, 24%, Fig. 1B, corresponding materials), suggesting that AML can infiltrate even incompletely developed scaffolds. This was also confirmed by Abarrategi et al. [6] who achieved hematopoietic cell engraftment in scaffolds with no calcification, although additional bone induction leads to improved engraftment. In our AML experiments, scaffold formation failure was rare – 2/88 (2%) scaffolds were completely resorbed, and 5/63 (8%) scaffolds were very small (< 10 mg). We, therefore, suggest that assessment of a material's BM formation can serve as a valuable predictor for achieving sufficiently large scaffolds with good reproducibility in further xenotransplantation experiments. We ultimately selected Sigma ECM gel and collagen‐HA fiber for their superior BM niche formation and injectability which simplified the protocol and the subsequent animal care. If pre‐seeding was required, we would opt for the solid collagen‐HA matrix that offered perfect reproducibility and could instantly soak up the medium with nearly homogeneous cell distribution across the matrix (data not shown).

We expected that biomaterials with larger BM niche cavities would yield more extracted AML cells, which was not true. We retrieved small numbers of AML cells from the scaffolds, similar to Abarrategi et al. and Mian et al., (0.1–1 × 10^6^) [6, 7], but much lower than Reinisch et al. (50 × 10^6^ cells) [27]. To increase the yields, we tested additional bone induction, that was also used in the aforementioned reports where Abarrategi et al. achieved higher AML engraftment with BMP‐2 added at implantation and Reinisch et al. described enlarged scaffolds with repeated administration of parathyroid hormone *in vivo* [6, 27]. We achieved formation of larger BM niche cavities and increased scaffold size with a BMP‐2 dose described for wound healing and bone regeneration (2.5 μg per scaffold) which was one log lower than in the previous scaffold protocol (50 μg) [6, 28, 29]. We could not assess the effect on AML cell yields as we only used BMP‐2 in the final experiment with non‐engrafting samples with generally low engraftment. However, the BMP‐2 addition offered a simple way to enhance scaffold formation. We speculate that a higher dose could further increase scaffold formation but at significantly higher costs.

We did not confirm any of the benefits—faster engraftment or engraftment of problematic samples—reported for the intra‐niche over the standard i.v. xenotransplantation route [6]. Moreover, the mix route provided minimum AML migration outside the AML‐seeded scaffolds, which represents a disadvantage if PB bleeding is used for engraftment monitoring or BM is used as an additional source of AML cells. Similar behavior was also observed in the original reports [6, 7]. With the i.v. route we were able to engraft 2/4 non‐engrafting AMLs and only 1/4 with the i.sc. route. Moreover, the intra‐niche delivery exhibited a so far unreported T‐cell expansion. We detected a low percentage of CD3^+^ cells with a well‐engrafting AML 0492 with mix condition, but a full‐fledged T‐cell expansion causing GVHD was encountered only with the non/low‐engrafting samples, suggesting that the T‐cells are normally outcompeted and/or suppressed by the AML graft. This occurred despite an OKT3 antibody pre‐treatment that is routinely and reliably applied in our laboratory for i.v. xenotransplantations [16]. The T‐cell expansion in scaffolds was not reported elsewhere, as most authors xenotransplanted samples with physically isolated AML cells or depleted T‐cells [4, 5, 27]. Abarrategi et al. used OKT3, however, with *in vitro* AML pre‐seeding [6]. An intra‐osseous injection of OKT3 pre‐treated AML cells (without scaffolds) was successfully used by de la Guardia et al. with a higher OKT3 dose of 5.83 μg per 1 × 10^6^ mononuclear cells, indicating that the OKT3 protocol might be usable for intra‐niche xenotransplantation but with an increased dose or staining time [30]. Taken together, the i.v. route provided the most robust AML engraftment. We speculate that intra‐niche delivery could provide higher engraftment for the poorly engrafting samples with improved T‐cell depletion, since T‐cell expansion is known to decrease AML engraftment [31].

Regarding general aspects, we did not see tumor formation in scaffolds. This was previously observed with calcium phosphate scaffolds [4, 5]. Here, we saw atypical enlargement with the ceramic β‐TCP granules, with 1/2 AML samples. Although this is not enough to confirm this effect, we speculate whether using calcium phosphate scaffolds might somehow induce focal tumor formation.

Sub‐lethal irradiation before AML xenotransplantation into pre‐established scaffolds has been employed in some [5, 27] but not all investigations [4, 5, 8]. Since the conditioning is dispensable for AML engraftment, even in murine models lacking scaffolds, we did not use it [31, 32, 33].

The comparison of the protocol compiled here with those already established highlights several significant points. (1) Our method eliminates an *in vitro* MSC or AML pre‐seeding step [11, 19, 34] or MSC differentiation priming [19, 34], thus reducing time and cost requirements. (2) Our approach is adaptable to any available biomaterial that can support BM niche formation, injectable or monolithic. (3) The protocol assembled here most closely resembles the one by Reinisch et al., with minor divergences, such as different ECM gel, BMP‐2 used instead of parathormone, and preferential i.v. xenotransplantation [9, 27].

## 5 Conclusions

In summary, we define a simple and modular approach for robust AML engraftment in humanized scaffolds. We used both proven and novel materials to demonstrate that biomaterial selection is not a limiting step and that surgical implantation can be avoided not only with ECM gels, but also with a material such as ground collagen, which represents a novel approach. Our data show that simple i.v. xenotransplantation is the most straightforward way to engraft even poorly engrafting samples. We believe that these findings will facilitate the adoption of scaffolds in hematological xenograft studies.

## Conflict of interest

The authors declare no conflict of interest.

## Author contributions

MSC donor enrolment—MR; AML patient enrolment—ZR, JM; Collagen biomaterials—LV; *In vitro* experiments—DB, ZH, JHyl, MC; Animal experiments—DB, ZH, MC; Histology—JV, FS, KM, LK; Molecular analyses—AF; Bioinformatics—JHyn; Study design—ZR, JM, MC; Manuscript preparation—MC; Manuscript revision—DB, ZH, JHyl, KM, AF, JHyn, ZR, JM. All authors read and approved the manuscript.

### Peer review

The peer review history for this article is available at https://www.webofscience.com/api/gateway/wos/peer‐review/10.1002/1878‐0261.13790.

## Supporting information


**Table S1.** Donor and patient characteristics.
**Table S3**. Bioinformatic analysis pipeline.
**Table S4**. Detected gene variants.
**Fig. S1**. The effect of mouse age on scaffold formation.
**Fig. S2**. Examples of biomaterials and extracted scaffolds.
**Fig. S3**. Comparison of collagen fibers with and without collagen crosslinking.
**Fig. S4**. Flow‐cytometric analysis – basic gating strategy.
**Fig. S5**. Immunophenotype examples for AML samples from Fig. 2.
**Fig. S6**. CD34+CD38‐ fraction assessed in samples from Fig. 2.
**Fig. S7**. Immunophenotype examples for AML samples from Fig. 3.
**Fig. S8**. CD34+CD38‐ fraction assessed in samples from Fig. 3.
**Fig. S9**. Amplicon analysis of WT1 mutations of samples from Fig. 3.

**Table S2.** Detailed experimental data.

## Data Availability

The data that support the findings of this study are available as supplementary material – Table S2. The bioinformatic pipeline for next‐generation sequencing data analysis is available in GitHub at https://github.com/Hynst/mix_mouse_human_pipeline.
