# Supplementary material for: Robust acute myeloid leukemia engraftment in humanized scaffolds using injectable biomaterials and intravenous xenotransplantation
Source: Mol Oncol. 2025 Jan 22;19(5):1371–85. doi: 10.1002/1878-0261.13790 (PMC12077274; doi:10.1002/1878-0261.13790)
Supplement: Supplementary file 1 — Table S1. Donor and patient characteristics. Table S3. Bioinformatic analysis pipeline. Table S4. Detected gene variants. Fig. S1. The effect of mouse age on scaffold formation. Fig. S2. Examples of biomaterials and extracted scaffolds. Fig. S3. Comparison of collagen fibers with and without collagen crosslinking. Fig. S4. Flow‐cytometric analysis – basic gating strategy. Fig. S5. Immunophenotype examples for AML samples from Fig. 2. Fig. S6. CD34+CD38‐ fraction assessed in samples from Fig. 2. Fig. S7. Immunophenotype examples for AML samples from Fig. 3. Fig. S8. CD34+CD38‐ fraction assessed in samples from Fig. 3. Fig. S9. Amplicon analysis of WT1 mutations of samples from Fig. 3. [file MOL2-19-1371-s002.docx]

**Robust acute myeloid leukemia engraftment in humanized scaffolds using injectable biomaterials and intravenous xenotransplantation**

**Supplementary tables**

**Table S1.** Donor and patient characteristics. f – female, ID – identifier, ELN 2007 – cytogenetic risk according to European LeukemiaNet classification(1), FAB – French-American-British classification, m – male, “-“ – not available, WHO 2008 – World Health Organization classification(2).

**Table S3.** Bioinformatic analysis pipeline. vcf – variant call format.

| **Software** | **Version** | **Step** |
| --- | --- | --- |
| UMI tools | 1.0.1 | read deduplication |
| cutadapt | 1.18 | adapter trimming |
| bwa | 0.7.17 | reads mapping |
| Bedtools | 2.24.0 | region reads coverage |
| GATK Mutect2 | gatk4=4.1.0.0 | variant calling |
| Bcftools | 1.8 | vcf processing and normalization |
| VEP | 96 | variant annotation |

**Table S4**. Detected gene variants.

| **Gene** | **Nucleotide change** | **Protein change** |
| --- | --- | --- |
| **AML 0244** | | |
| *ASXL1* | NM_015338.6:c.3943C>T | NP_056153.2:p.(Gln1315*) |
| *DNMT3A* | NM_022552.5:c.2644C>T | NP_072046.2:p.(Arg882Cys) |
| *TET2* | NM_001127208.3:c.4997dup | NP_001120680.1:p.(Leu1667Serfs*3) |
| *U2AF1* | NM_006758.3:c.101C>T | NP_006749.1:p.(Ser34Phe) |
| **AML 1709** | | |
| *DNMT3A* | NM_022552.5:c.2018G>A | NP_072046.2:p.(Gly673Asp) |
| *IDH2* | NM_002168.4:c.419G>A | NP_002159.2:p.(Arg140Gln) |
| *NRAS* | NM_002524.5:c.181C>A | NP_002515.1:p.(Gln61Lys) |
| **AML 0492** | | |
| *IDH1* | NM_005896.3:c.394C>T | NP_005887.2:p.(Arg132Cys) |
| *KRAS* | NM_004985.4:c.38G>A | NP_004976.2:p.(Gly13Asp) |
| *WT1* #1 | NM_000378.6:c.1094_1097dup | NP_000369.4:p.(Val367Serfs*7) |
| *WT1* #2 | NM_000378.6:c.1107_1108insAACGGTCG | NP_000369.4:p.(Ala370Asnfs*70) |
| *WT1* #3 | NM_000378.6:c.1074dup | NP_000369.4:p.(Val359Cysfs*14) |
| *WT1* #4 | NM_000378.6:c.1093_1102dup | NP_000369.4:p.(Arg368HisfsTer8) |

**Supplementary figures**

**Fig. S1.** The effect of mouse age on scaffold formation. Only the data from experiments on scaffold creation without AML xenotransplantation are shown (from Fig. 1B,D and Fig. 4B,E).

**
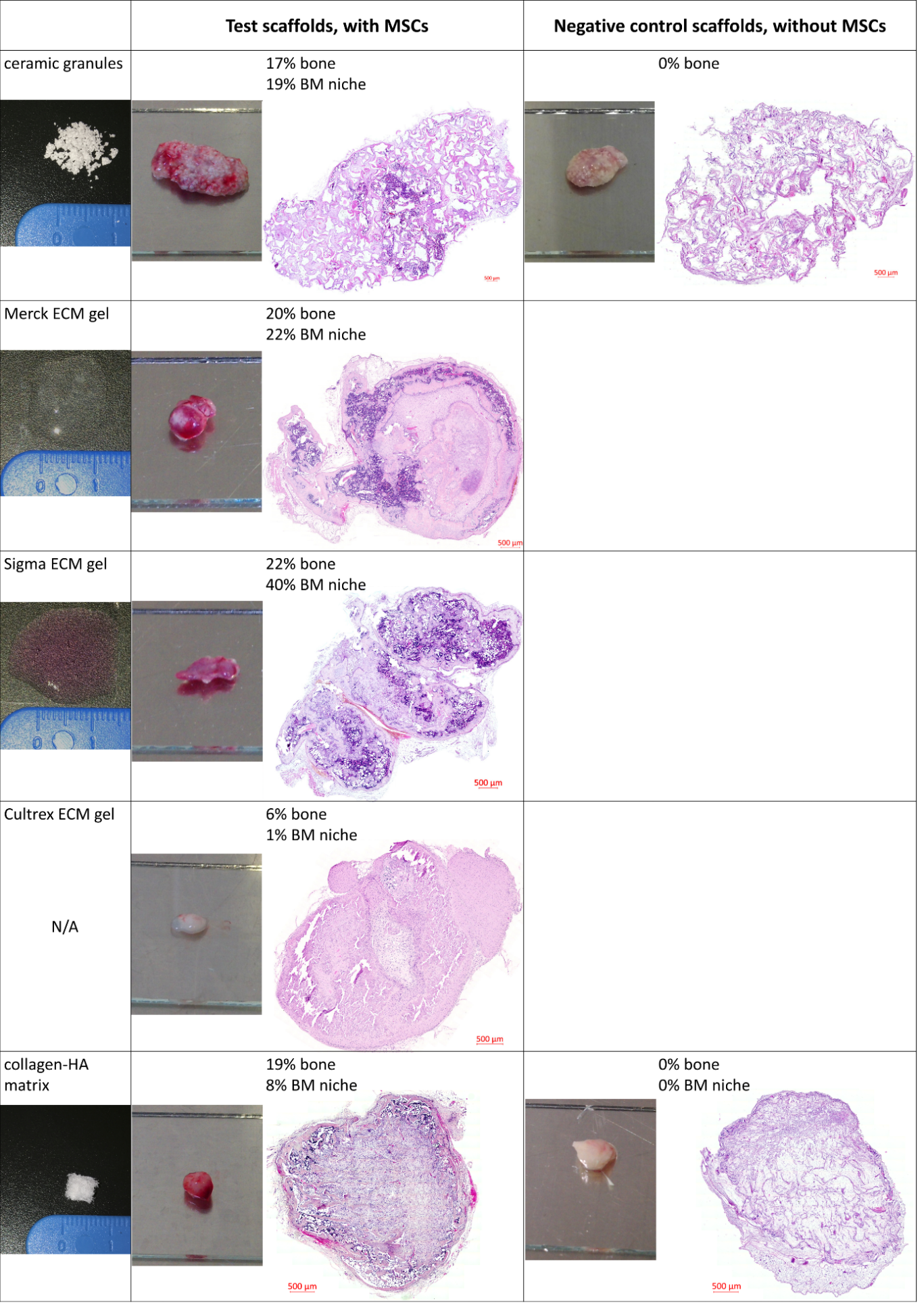

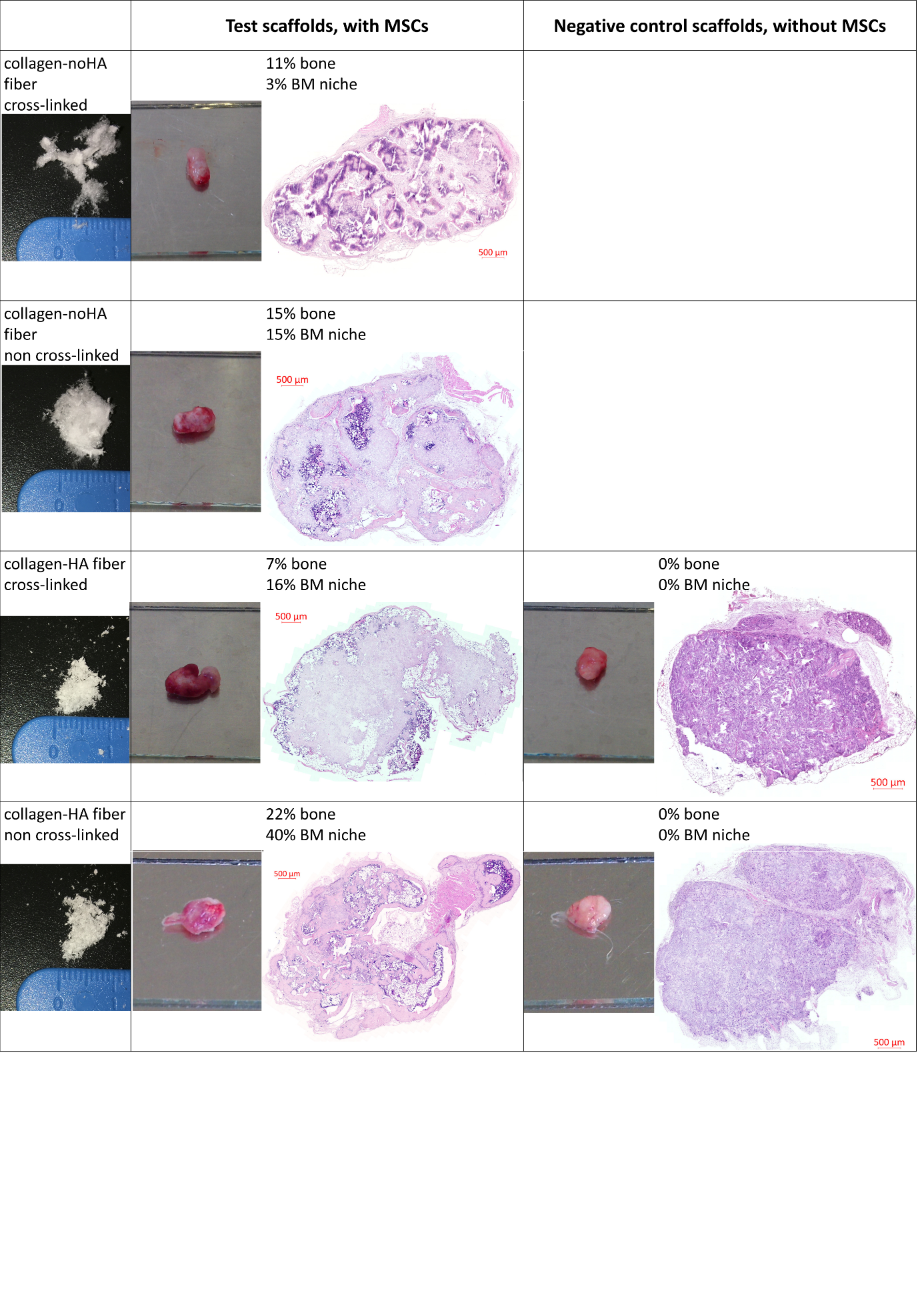
**

**Fig. S2.** Examples of biomaterials and extracted scaffolds. From the left: photographs of the biomaterials pre-implantation (enclosed ruler with mm scale); photographs and histology slides in hematoxylin-eosin staining from scaffolds extracted 8 weeks post-implantation. Test scaffolds were implanted with 1.5-2.0 × 10^6 second passage MSCs (Fig. 1C), negative control scaffolds (if available) were implanted without MSCs. The collagen-HA matrix is shown also in Fig. 1C. BM – bone marrow, ECM – extracellular matrix, HA – hydroxyapatite, MSC – mesenchymal stromal cells.

**Fig. S3.** Comparison of collagen fibers with and without collagen crosslinking. In regards to bone and bone marrow formation in scaffolds. Area of mature bone trabeculae and hematopoiesis quantified using image analysis of histological slides. Positive case defined as ≥ 0.1% of area. Scaffolds generated with 2 × 10^6 second passage BM MSCs. Columns in graphs show mean values with standard deviation. No statistical differences found (unpaired non-parametric t-test). HA – hydroxyapatite, hBM MSC – human bone marrow mesenchymal stromal cells.

**Fig. S4.** Flow-cytometric analysis – basic gating strategy. Upper row: First, cells were selected to eliminate debris. Then, live cells were selected, followed by a selection of singlets. Next, human cells were selected for further immunophenotype analysis (performed only in murine samples). Lower row: Basic populations were gated based on SSC-A vs hCD45 aided with overlay of CD34, CD14, CD33, and CD3 expression. In some xenografts, as the one shown here, the blast and monocyte populations were difficult to distinguish. The blast gate was thus set to contain mostly CD34+CD14- cells, while the monocyte gate was set to include CD34-CD14+ cells. The granulocyte gate was set to contain the remaining mature cells with higher SSC in general. CD34 expression was gated for strictly positive and CD38 for strictly negative cells. The primitive CD34+CD38- cells were obtained from the intersection of CD34+ and CD38- together with blast gate. CD14+ gate contained strictly positive cells, and CD33+ contained also dim cells. Sample shown – AML 0413, scaffold from an intra-venously (i.v) injected mouse. h – human, m – murine, SSC – side scatter.

**Fig. S5.** Immunophenotype examples for AML samples from Fig. 2. Upper row (person icon) shows the original primary sample, and the lower row (mouse icon) shows the corresponding xenograft sample. For AML 0244 (**A**), the xenografts carried mainly a blast and a monocyte population. AML 1709 (**B**) xenografts carried only a blast population. Neither sample showed signs of concurrent healthy hematopoiesis engraftment. The AML engraftment was thus simply assessed as hCD45+Lym- cells, as not all blast/monocyte cells expressed CD34 or CD33. AML – acute myeloid leukemia, ECM – extracellular matrix, h – human, NA – not available.

**Fig. S6.** CD34+CD38- fraction assessed in samples from Fig. 2 – comparison of AML engraftment in different biomaterials (within-between two way ANOVA, unpaired non-parametric t-test). Columns in graphs show mean values. AML – acute myeloid leukemia, BM – bone marrow, col.– collagen, ctrl – control, ECM – extracellular matrix, ns – not significant, PB – peripheral blood, scaff – scaffold, * P < 0.05.

**Fig. S7.** continued.

**Fig. S7.** Immunophenotype examples for AML samples from Fig. 3. Upper row (person icon) shows the original primary sample, and the lower rows (mouse icon) show the corresponding xenograft samples. AML 0244 xenografts (**A**) carried mainly a blast and a monocyte population, a small CD3+ lymphocyte population in some mice. AML 1709 xenografts (**B**) carried a dominant blast population, some mice xenotransplanted with the mix route also showed a concurrent CD3+ T-cell expansion. Neither sample showed signs of concurrent healthy hematopoiesis engraftment. The AML engraftment was thus simply assessed as hCD45+Lym- cells, as not all blast/monocyte cells expressed CD34 or CD33. AML – acute myeloid leukemia, ECM – extracellular matrix, i.v. intra-venous, NA – not available.

**Fig. S8.** CD34+CD38- fraction assessed in samples from Fig. 3 – comparison of different xenotransplantation routes (within-between two way ANOVA, unpaired or paired non-parametric t-test).
AML – acute myeloid leukemia, BM – bone marrow, ctrl – control, i.sc. – intra-scaffold, i.v. – intravenous, ns – not significant, PB – peripheral blood, scaff – scaffold, * P < 0.05, ** P < 0.01.

**Fig. S9.** Amplicon analysis of *WT1* mutations of samples from Fig. 3. The method sensitivity was 0.1 % variant allele frequency. Altogether, 4 different *WT1* mutations were detected. Three of 4 mutations were detected in the original AML sample (on the left). Mice were analyzed individually. One symbol per column means that a mutation was found only in one mouse of a group. Two identical symbols per column mean that the same mutation was found in two mice of a group. AML – acute myeloid leukemia, BM – bone marrow, ctrl – control, i.sc. – intra-scaffold, i.v. – intravenous, n – number of mice per group, NA – not analyzed, PB – peripheral blood, S – scaffold, VAF – variant allele frequency.

**References**

1. Döhner H, Estey E, Grimwade D, Amadori S, Appelbaum FR, Büchner T, et al. Diagnosis and management of AML in adults: 2017 ELN recommendations from an international expert panel. Blood. 2017 Jan 26;129(4):424–47.

2. Vardiman JW, Thiele J, Arber DA, Brunning RD, Borowitz MJ, Porwit A, et al. The 2008 revision of the World Health Organization (WHO) classification of myeloid neoplasms and acute leukemia: rationale and important changes. Blood. 2009 Jul 30;114(5):937–51.
